# Ternary Organic Solar Cells Based on a Wide-Bandgap Polymer with Enhanced Power Conversion Efficiencies

**DOI:** 10.1038/s41598-019-48306-x

**Published:** 2019-08-19

**Authors:** Hyeongjin Hwang, Dong Hun Sin, Chaneui Park, Kilwon Cho

**Affiliations:** Department of Chemical Engineering, Pohang University of Science and Technology, Pohang, 37673 Korea

**Keywords:** Solar cells, Electronic devices, Solar cells, Electronic devices, Solar cells

## Abstract

A low-bandgap acceptor (ITIC) was added to a binary system composed of a wide-bandgap polymer (PBT-OTT) and an acceptor (PC_71_BM) to increase the light harvesting efficiency of the associated organic solar cells (OSCs). A ternary blend OSC with an acceptor ratio of PC_71_BM:ITIC = 8:2 was found to exhibit a power conversion efficiency of 8.18%, which is 18% higher than that of the binary OSC without ITIC. This improvement is mainly due to the enhanced light absorption and optimized film morphology that result from ITIC addition. Furthermore, an energy level cascade forms in the blend that ensures efficient charge transfer, and bimolecular and trap-assisted recombination is suppressed. Thus the use of ternary blend systems provides an effective strategy for the development of efficient single-junction OSCs.

## Introduction

Organic solar cells (OSCs) can be lightweight, flexible, transparent, and mass-producible^1–3^. Recent studies have reported single-junction OSCs with significantly increased power conversion efficiencies (PCEs) > 10%^4–8^.

In a general approach to the fabrication of OSCs, the photoactive layer can be prepared by mixing a light-harvesting polymer as a donor and an electron-accepting fullerene derivative as an acceptor. However, such binary OSCs have relatively narrow light absorption windows, which restricts their photocurrent generation^9,10^. In order to increase their light absorption, tandem structures have been introduced. A bottom cell based on a wide-bandgap polymer and a top cell based on a narrow-bandgap polymer are linked in series, which results in complementary absorption of the solar spectrum and boosts the power conversion efficiency of the incorporated cells^11^. However, tandem structures have several drawbacks such as their complex fabrication process and high production costs, which limit their practical applications^12^.

In the past few years, ternary blend OSCs have been developed that exhibit extended light absorption and do not require complicated fabrication processes^13–15^. The light absorption spectrum of the third component is generally complementary to that of the light-harvesting polymer and is introduced into the donor/acceptor binary blend^10^. The presence of the third component can result in the formation in combination with the other two components of an energy level cascade for charge transfer, and can also enhance the development of the film morphology. Furthermore, ternary single-junction OSCs can be fabricated with a process that is simpler than the complex processes required for the fabrication of tandem OSCs^16–18^.

Recently, 3,9-bis(2-methylene-(3-(1,1-dicyanomethylene)-indanone))-5,5,11,11-tetrakis(4-hexylphenyl)-dithieno[2,3-d:2′,3′-d′]-s-indaceno[1,2-b:5,6-b′]dithiophene) (ITIC) was developed as a narrow-bandgap acceptor. ITIC exhibits strong light absorption in the infrared region and its energy level can be adjusted for compatibility with other absorbing materials, so ITIC-based OSCs have been found to exhibit outstanding performances^19–25^. However, ITIC exhibits high photovoltaic performance in combination with only very few polymers because its aggregation properties pose difficulties for the control of the film morphologies of ITIC-based blend films. Moreover, in some cases, ITIC-based OSCs exhibit relatively low fill factors (FFs) because of recombination losses and low electron mobilities^26^.

For ITIC to act as an efficient acceptor, it is important to control its aggregation^27^. Such control can be achieved by mixing ITIC with [6,6]-phenyl-C_71_-butyric acid methyl ester (PC_71_BM), which has high miscibility with donor polymers. The resulting mixed acceptor can then act as a light harvester and be miscible with donor polymers without severe aggregation.

In this study, we combined this mixed acceptor based on the narrow-bandgap ITIC and PC_71_BM with a wide-bandgap polymer, PBT-OTT^28^, and sought to optimize the light absorption and morphology of the resulting photoactive layer. The absorption of ITIC complements that of PBT-OTT (300 ≤ *λ* ≤ 800 nm) and forms an energy cascade that promotes charge transfer in the ternary blend. Here, we define [ITIC] as the ITIC content (wt/wt) relative to that of PC_71_BM. [ITIC] was systematically varied from 0 to 100%. For [ITIC] = 20%, ITIC is well-mixed with PBT-OTT and PC_71_BM, which results in an optimized film morphology and a ternary-blend-based OSC with a PCE of 8.18%, which is 18% higher than that of the binary-blend-based OSC. The charge generation, charge transport, and recombination dynamics of the OSC were characterized to determine the effects of the use of the ternary blend. These results demonstrate that the ternary blend approach is an effective strategy that enables the simple fabrication of highly efficient OSCs.

## Results and Discussion

### Optoelectric properties and the charge transfer mechanism

**Figure 1:**
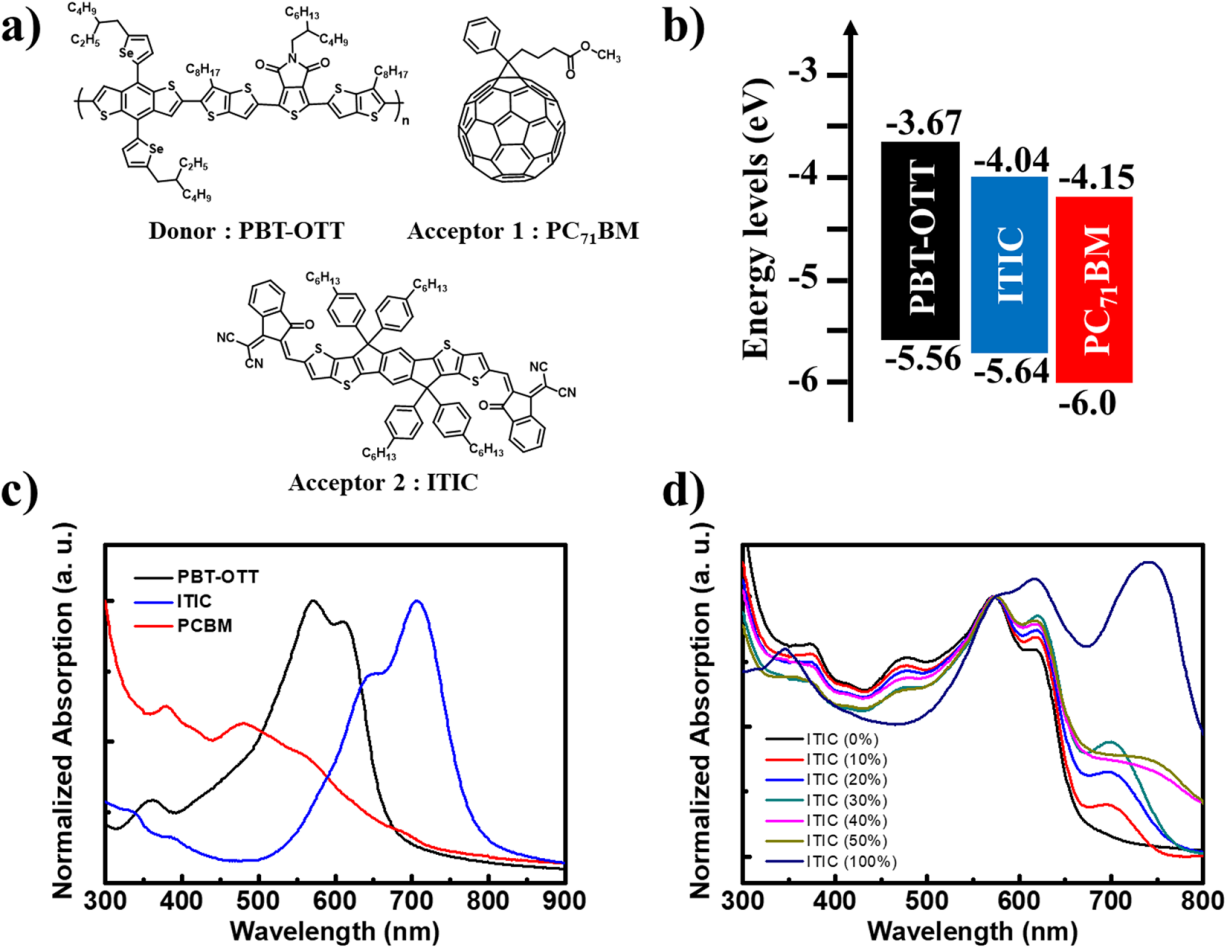
(**a**) Chemical structures of PBT-OTT, PC_71_BM, and ITIC. (**b**) Energy levels diagrams for PBT-OTT, ITIC, PC_71_BM. (**c**) UV-Vis absorption spectra of PBT-OTT, ITIC, and PC_71_BM films. (**d**) UV-Vis absorption spectra of PBT-OTT:ITIC:PC_71_BM with different ITIC contents (wt %); the number following “ITIC” in the legend represents the percentage of ITIC used in blend films.

The chemical structures of PBT-OTT, PC_71_BM, and ITIC are presented in Fig. 1a. The highest occupied molecular orbital (HOMO) energy levels of PBT-OTT and ITIC were determined from their onset oxidation potentials measured by cyclic voltammetry (CV), and their lowest unoccupied molecular orbital (LUMO) energy levels were determined from their optical bandgaps (Fig. 1b)^29^. The ternary blends provide broad and strong absorption covering the range of wavelengths from the visible to the near-infrared (Fig. 1c). The maximum absorptions of the PBT-OTT and ITIC films are at λ = 512 nm and 706 nm respectively. As the ITIC content of the PBT-OTT:PC_71_BM blend increases, the intensity of absorption in the range 680 ≤ λ ≤ 760 nm increases while that in the range 340 ≤ λ ≤ 510 nm decreases and the intensity of the PBT-OTT shoulder peak adjacent to the maximum absorption peak in the film state strengthens.

**Figure 2:**
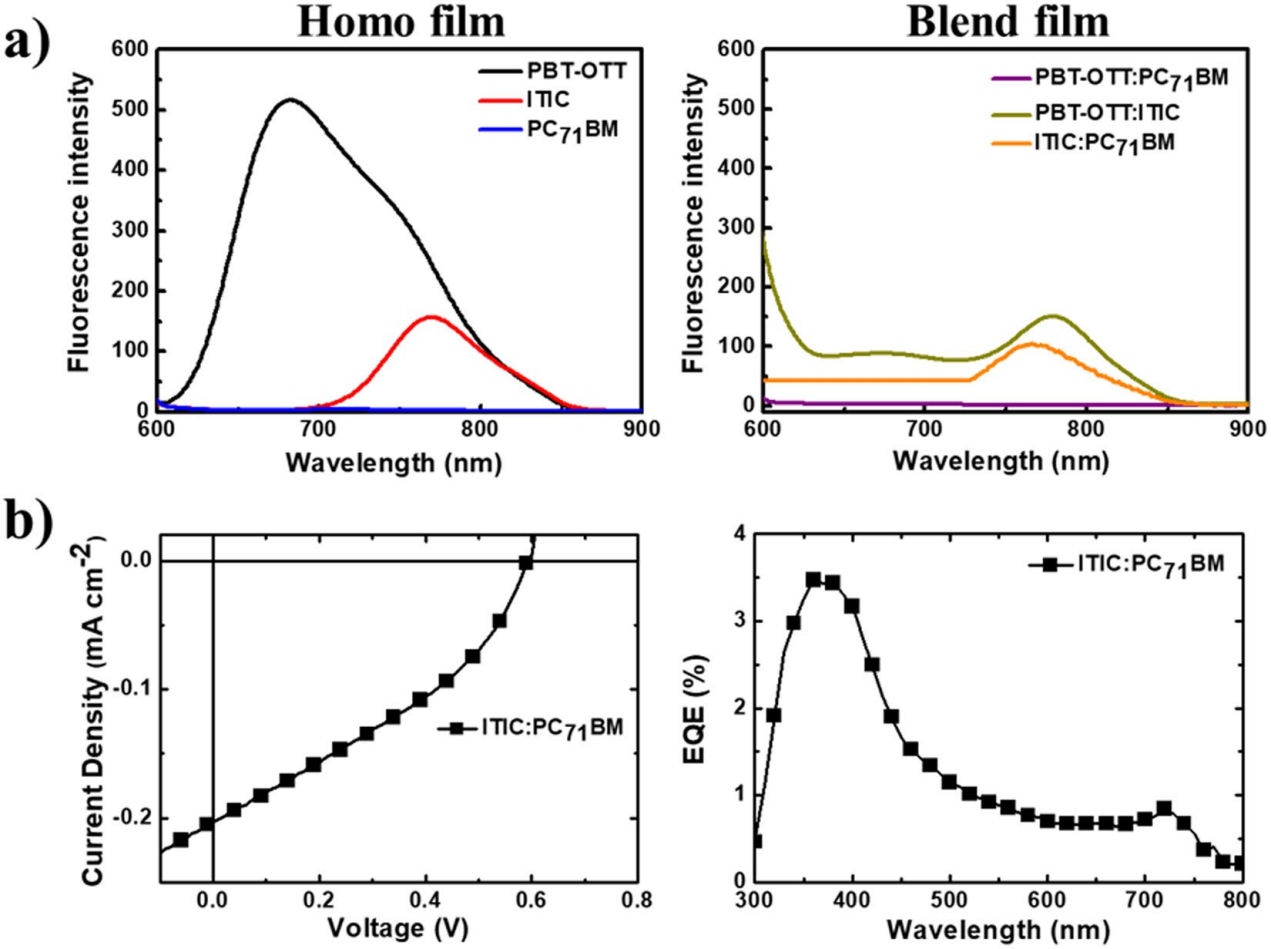
(**a**) Photoluminescence spectra of PBT-OTT, ITIC, PC_71_BM, PBT-OTT:ITIC (1:1), PBT-OTT:PC_71_BM (1:1) excited at 570 nm, and ITIC:PC_71_BM (1:1) excited at 705 nm, and (**b**) photovoltaic properties of the ITIC:PC_71_BM binary device.

**Figure 3:**
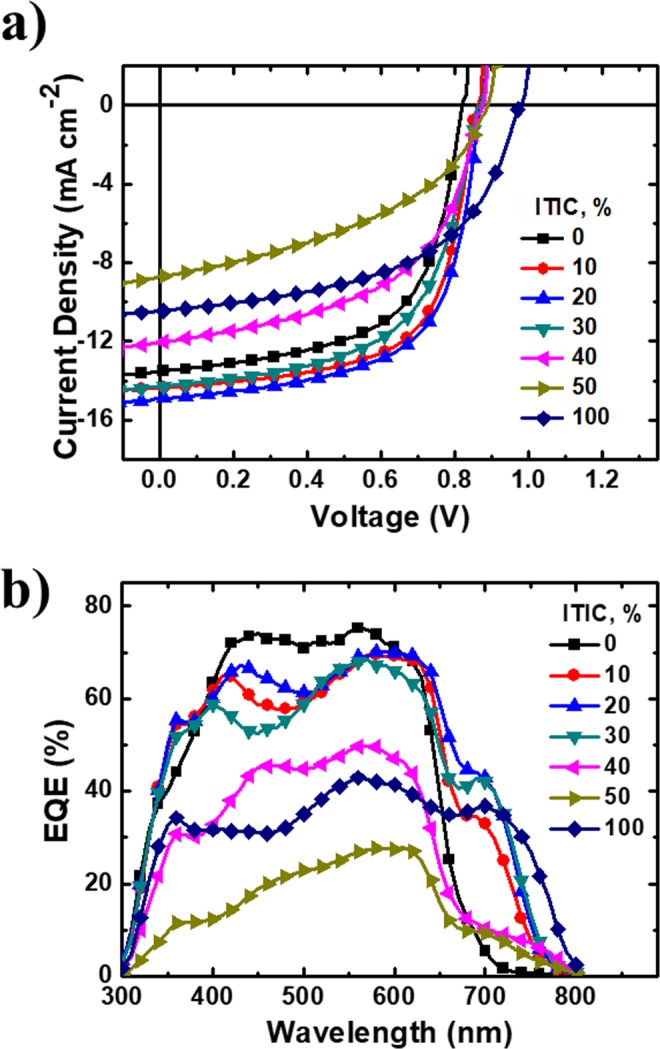
Photovoltaic performance of the ternary devices. (**a**) Characteristic* J-V* curves of the devices based on PBT-OTT:ITIC:PC_71_BM with different ITIC weight ratio under illumination of AM 1.5 G, 100 mW/cm^2^ light. (**b**) EQE curves of the ternary devices.

The presence of the ITIC acceptor results in cascaded energy levels, in contrast to those of the PBT-OTT:PC_71_BM blend (Fig. 1b). The LUMO energy level of ITIC is positioned between that of PBT-OTT and PC_71_BM. Cascaded LUMO energy level alignment promotes electron transfer between the components of the bulk heterojunction blend and ensures efficient exciton splitting and charge transport to the electrodes^30^. The HOMO energy level of ITIC lies between that of PBT-OTT and PC_71_BM, so holes are extracted efficiently from PC_71_BM. To demonstrate that the energy levels of the three components are cascaded, we obtained the film photoluminescence (PL) spectra of PBT-OTT, ITIC, PC_71_BM, PBT-OTT:ITIC (1:1), and PBT-OTT:PC_71_BM (1:1) with excitation at 570 nm, which corresponds to the maximum absorption of PBT-OTT, and of ITIC:PC_71_BM (1:1) with excitation at 705 nm, which corresponds to the maximum absorption of ITIC (Fig. 2). The emission of PBT-OTT is quenched without an increase in the ITIC PL signal and quenched completely without an increase in the PC_71_BM PL signal (Fig. 2a). These results confirm that photoinduced electrons can be transferred from PBT-OTT to ITIC and then to PC_71_BM. OSC devices based on the ITIC:PC_71_BM blend were found to exhibit photodiode characteristics in their *J-V* curves and EQE peaks in the ranges 300 ≤ *λ* ≤ 450 nm and 700 ≤ *λ* ≤ 800 nm, which correspond to the absorption ranges of PC_71_BM and ITIC respectively. These results reveal that holes and electrons transfer from PC_71_BM to ITIC and from ITIC to PC_71_BM respectively. Further, considering that there is evidence for hole transfer from ITIC to PBT-OTT in the demonstration of a PCE of 5.43% for a device based on PBT-OTT:ITIC (Table 1) and also that the EQE increases in the range 700 ≤ *λ* ≤ 800 nm (Fig. 3b) because ITIC exhibits high absorption in the PBT-OTT:ITIC blend (1:1) (Fig. 1c), we conclude that holes can transfer from PC_71_BM to ITIC and finally to PBT-OTT. Thus, an energy level cascade forms in the ternary blend system.

**Table 1:** Detailed photovoltaic parameters of PBT-OTT:ITIC:PC_71_BM based devices with different ITIC contents (wt%).

PBT-OTT:ITIC:PC_71_BM	ITIC:PC_71_BM [wt%]	*J*_*SC*_ [mA cm^−2^]	*V*_*OC*_ [V]	FF [%]	PCE_max_ (PCE_avg_)* [%]
1:0:1.5	0: 100	13.3 ± 0.3	0.83 ± 0.01	58.5 ± 3.2	6.74 (6.41 ± 0.43)
1:0.15:1.35	10: 90	13.9 ± 0.5	0.86 ± 0.01	63.6 ± 1.4	7.92 (7.59 ± 0.28)
1:0.3:1.2	20: 80	14.8 ± 0.2	0.87 ± 0.01	63.0 ± 1.2	8.18 (8.06 ± 0.14)
1:0.45:1.05	30: 70	14.5 ± 0.3	0.87 ± −0.1	55.5 ± 1.8	7.24 (7.02 ± 0.22)
1:0.6:0.9	40: 60	11.5 ± 0.7	0.88 ± 0.1	53.3 ± 2.0	5.60 (5.36 ± 0.24)
1:0.75:0.75	50: 50	8.28 ± 0.5	0.89 ± 0.1	42.1 ± 2.1	3.31 (3.08 ± 0.21)
1:1.5:0	100: 0	10.27 ± 0.4	0.97 ± 0.1	51.0 ± 2.1	5.43 (5.10 ± 0.36)

*The values in parentheses stand for the average PCEs with standard deviations from over 12 devices.

### Photovoltaic properties

The photovoltaic efficiencies of ternary blend OSCs fabricated with the inverted structure ITO/ZnO/active layer (PBT-OTT:ITIC:PC_71_BM)/MoOx/Al were evaluated. The overall donor-to-acceptor ratio in the active layer was fixed at 1:1.5 (wt/wt%). 3 vol % 1,8-diiodooctane (DIO) was used as a processing additive. In these ternary blends, the PC_71_BM:ITIC ratio was varied: [ITIC] = 10, 20, 30, 40, 50, and 100 wt %. Figure 3a shows the current density (*J*) vs. voltage (*V*) characteristics of the OSCs with the different ITIC contents, and their photovoltaic parameters are summarized in Table 1. The binary reference device based on PBT-OTT:PC_71_BM was found to exhibit the following characteristics: PCE = 6.74% with *J*_SC_ = 13.53 mA cm^−2^, *V*_OC_ = 0.82 V, and FF = 60.7%. For [ITIC] = 10%, the PCE increases to 7.92%, *J*_SC_ to 14.34 mA cm^−2^, *V*_OC_ to 0.86 V, and FF to 64.49%. For [ITIC] = 20% the photovoltaic characteristics are optimal: PCE = 8.18%, *J*_SC_ = 14.93 mA cm^−2^, *V*_OC_ = 0.87 V, and FF = 63.0%. These increases could be due to the expanded light absorption at wavelengths up to *λ* = 800 nm. For [ITIC] > 20%, the FF values decrease gradually; this trend is possibly due to a reduction of hole mobility measured by the space charge limited current (SCLC) method as [ITIC] increases (Fig. S1 and Table S2). For [ITIC] = 20%, the hole mobility *μ*_h_ is higher and *μ*_h_/*μ*_e_ is close to 1 (Table S2). However, for [ITIC] = 50%, *μ*_h_ is reduced and *μ*_h_/*μ*_e_ deviates from 1. We conclude that under the optimal conditions when [ITIC] = 20%, photo-generated charge carriers are extracted more efficiently to the electrodes in the device than at other [ITIC]. These phenomena are also affected by bimolecular recombination, which is discussed in Section 2.4. For [ITIC] > 20%, increases in the [ITIC] of the blend up to 50% simultaneously degrade the *J*_SC_ and FF values of the associated OSCs. *V*_OC_ gradually increases as [ITIC] increases, possibly because the high-lying LUMO of ITIC leads to charge transfer (CT) state with higher energy than that of PC_71_BM at the donor polymer/acceptor molecule interface. In ternary blends, CT states can form at both PBT-OTT/ITIC and PBT-OTT/PCBM interface, and average CT energy determines final *V*_OC_.

The overall EQEs for [ITIC] = 10, 20, and 30% in the ternary blend are significantly higher than those of the PBT-OTT:PC_71_BM binary blend (Fig. 3b). This result demonstrates that more photogenerated excitons in the active layer dissociate to free charges and are collected by the electrodes, as indicated by the significant increase in EQE for the range 630 ≤ *λ* ≤ 800 nm due to the increase in light absorption that results from the introduction of ITIC, and that the energy level cascade of PBT-OTT, ITIC, and PC_71_BM improves the charge carrier transport.

### Charge generation and dissociation dynamics

**Figure 4:**
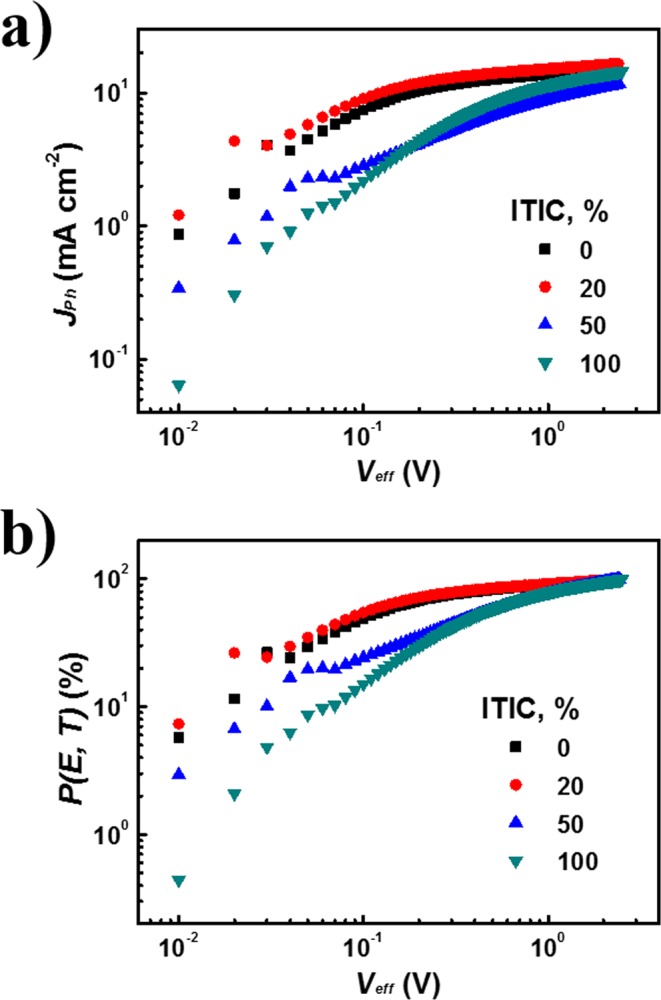
(**a**) Photocurrent density (*J*_*ph*_) versus effective voltage (*V*_*eff*_) characteristics. (**b**) *P(E, T)* versus *V*_*eff*_, where *P(E, T)* determined by normalized *J*_*ph*_ with *J*_*sat*_ (*J*_*ph*_/*J*_*sat*_).

To investigate the improved *J*_SC_ of the ternary OSCs, the charge generation and dissociation of the OSCs with [ITIC] = 0, 20, 50, and 100% were assessed by determining the saturation current density *J*_sat_ and the charge dissociation probabilities *P*(*E*, *T*). Figure 4a shows the photocurrent density (*J*_ph_) versus effective voltage (*V*_eff_) curves for these ternary devices. Here, *J*_ph_ is defined as *J*_ph_ = *J*_L_ − *J*_D_, where *J*_L_ and *J*_D_ are the photocurrent densities under illumination and in the dark respectively. *V*_eff_ is defined as *V*_eff_ = *V*_0_ − *V*_a_, where *V*_0_ is the voltage at which *J*_ph_ is zero and *V*_a_ is the applied bias voltage^31^. Generally, all photogenerated excitons are assumed to dissociate into free charge carriers at high *V*_eff_ (approximately 2 V), and then *J*_sat_ is only limited by the maximum exciton generation rate (*G*_max_). As a result, *J*_sat_ equals to *qLG*_max_, where *q* is the constant of the elementary charge and *L* is the active layer thickness^32^. *G*_*max*_ rises as [ITIC] rises to 20%: for [ITIC] = 0%, for *G*_*max*_ = 9.51 × 10^27^ m^−3^ s^−1^ and for *J*_*sat*_ = 152.3 Am^−2^; for [ITIC] = 20%, for *G*_*max*_ = 10.31 × ^27^ m^−3^ s^−1^ and for *J*_*sat*_ = 165.1 Am^−2^; for [ITIC] = 50%, for *G*_*max*_ = 7.29 × 10^27^ m^−3^ s^−1^ and for *J*_*sat*_ = 116.8 Am^−2^; for [ITIC] = 100%, for *G*_*max*_ = 9.08 × 10^27^ m^−3^ s^−1^ and for *J*_*sat*_ = 145.4 A m^−2^. The increase in *G*_max_ with [ITIC] suggests that the overall absorption and exciton generation increase in the ternary blend OSCs resulted from the complementary absorption that arose from the loading of a small amount of ITIC and the resulting energy cascade of PBT-OTT:ITIC:PC_71_BM. By normalizing *J*_ph_ with *J*_sat_*, P*(*E, T*) can be calculated (Fig. 4b)^33^. *P*(*E, T*) is highest for [ITIC] = 20%; for [ITIC] = 0%, *P*(*E, T*) = 88.7; for [ITIC] = 20%, *P*(*E, T*) = 90.5; for [ITIC] = 50%, *P*(*E, T*) = 74.7; for [ITIC] = 100%, *P*(*E, T*) = 79.5. This trend indicates that the incorporation of low levels of ITIC increases the exciton dissociation at the donor/acceptor interfaces and ensures sufficient charge transport and collection at the electrodes in the active layer.

### Charge recombination dynamics

**Figure 5:**
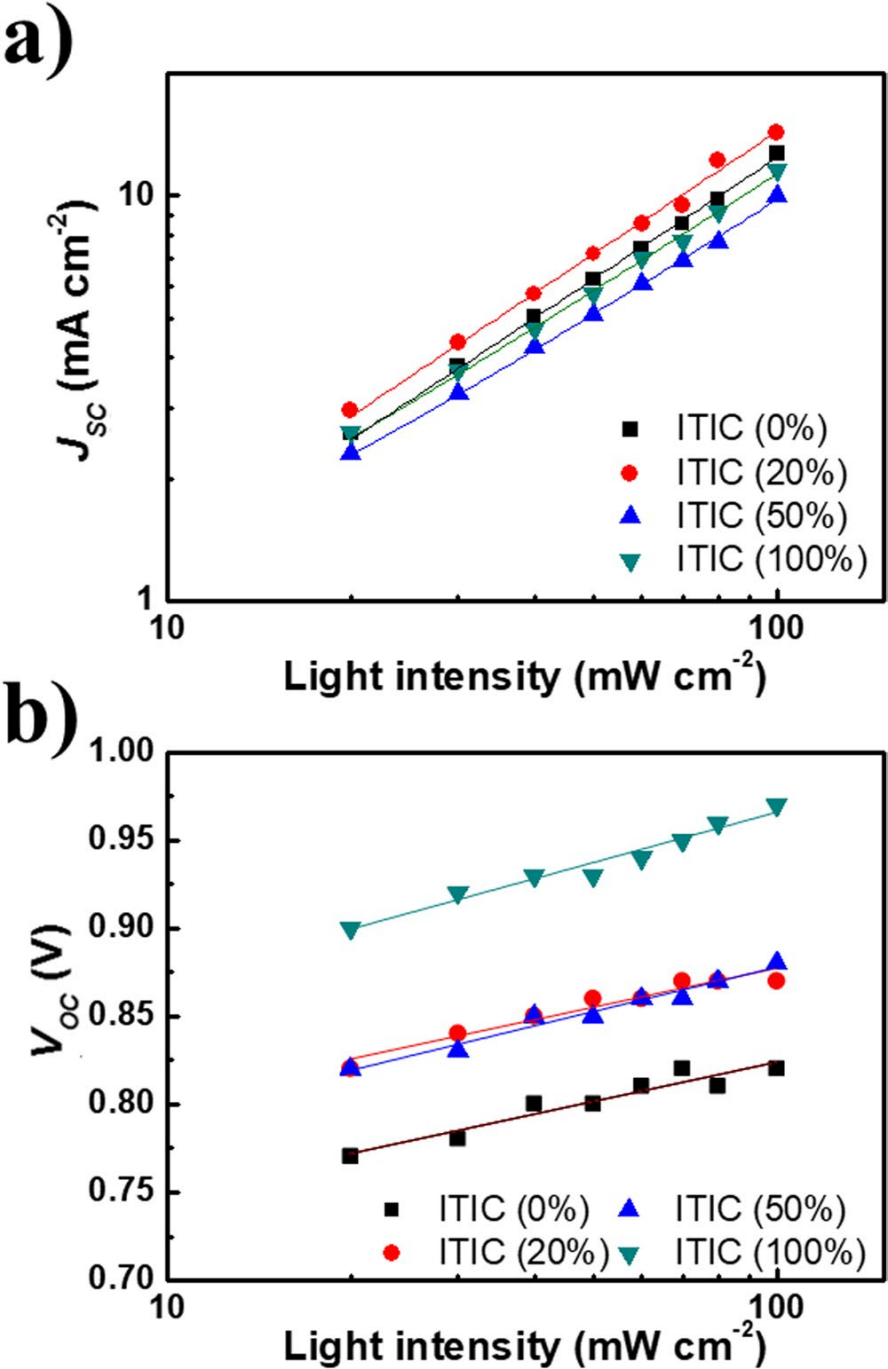
(**a**) Short-circuit current density (*J*_*sc*_) versus light intensity and (**b**) open-circuit voltage (*V*_*oc*_) versus light intensity for the ternary system.

To further investigate the effects of charge recombination dynamics on the efficiencies of the ternary OSCs, we obtained the *J*_SC_-light illumination intensity plots for the four devices (Fig. 5a). It is known that *J*_SC_ has a power-law dependence on the light intensity (*P*_light_), i.e. *J*_SC_ $\propto$ (*P*_light_)^S^, in OSCs^34^. In these devices, weak bimolecular recombination gives rise to *S* ≈ 1. At low ITIC concentrations, ITIC has little effect on bimolecular recombination i.e., for [ITIC] = 0%, (the host binary blend, PBT-OTT:PC_71_BM), *S* = 0.98 and at [ITIC] = 20%, *S* = 0.99, but for [ITIC] = 50%, *S* = 0.90 and for [ITIC] = 100%, *S* = 0.91; thus bimolecular recombination increases for ITIC concentrations above 20%. This trend is correlated with that for the films with aggregated morphologies, which is discussed in the following section. Increases in bimolecular recombination for [ITIC] > 20% are also related to the decreases in the *J*_SC_ and FF of the associated cells. Further, the bimolecular recombination trend is well correlated with those in *μ*_h_ and the ratio of *μ*_h_ to *μ*_e_ obtained using SCLC for the four devices.

The relationship between *V*_OC_ and *P*_light_ in OSCs with various [ITIC] is presented in Fig. 5b. The slope of each *V*_OC_ versus ln(*P*_light_) plot can be used to investigate the extent of trap-assisted recombination in the OSCs: a slope of *k*_B_*T*/*q* indicates whether trap-assisted recombination is dominant or not, where *k*_B_ is Boltzmann’s constant, *T* is the absolute temperature. For Shockley-Read-Hall recombination or trap-assisted, the dependence of *V*_OC_ on *P*_light_ is stronger and results in the slope of 2*k*_B_*T*/*q*^34,35^. In our case, the blend with [ITIC] = 20% produces the smallest slope, 1.44 *k*_B_*T*/*q*. These results show that the incorporation of a low concentration of ITIC in the host blend reduces the density of interfacial surface traps in the active layer; this reduction suppresses trap-assisted recombination and contributes to an increase in *J*_SC_.

### Thin film morphology and molecular ordering

**Figure 6:**
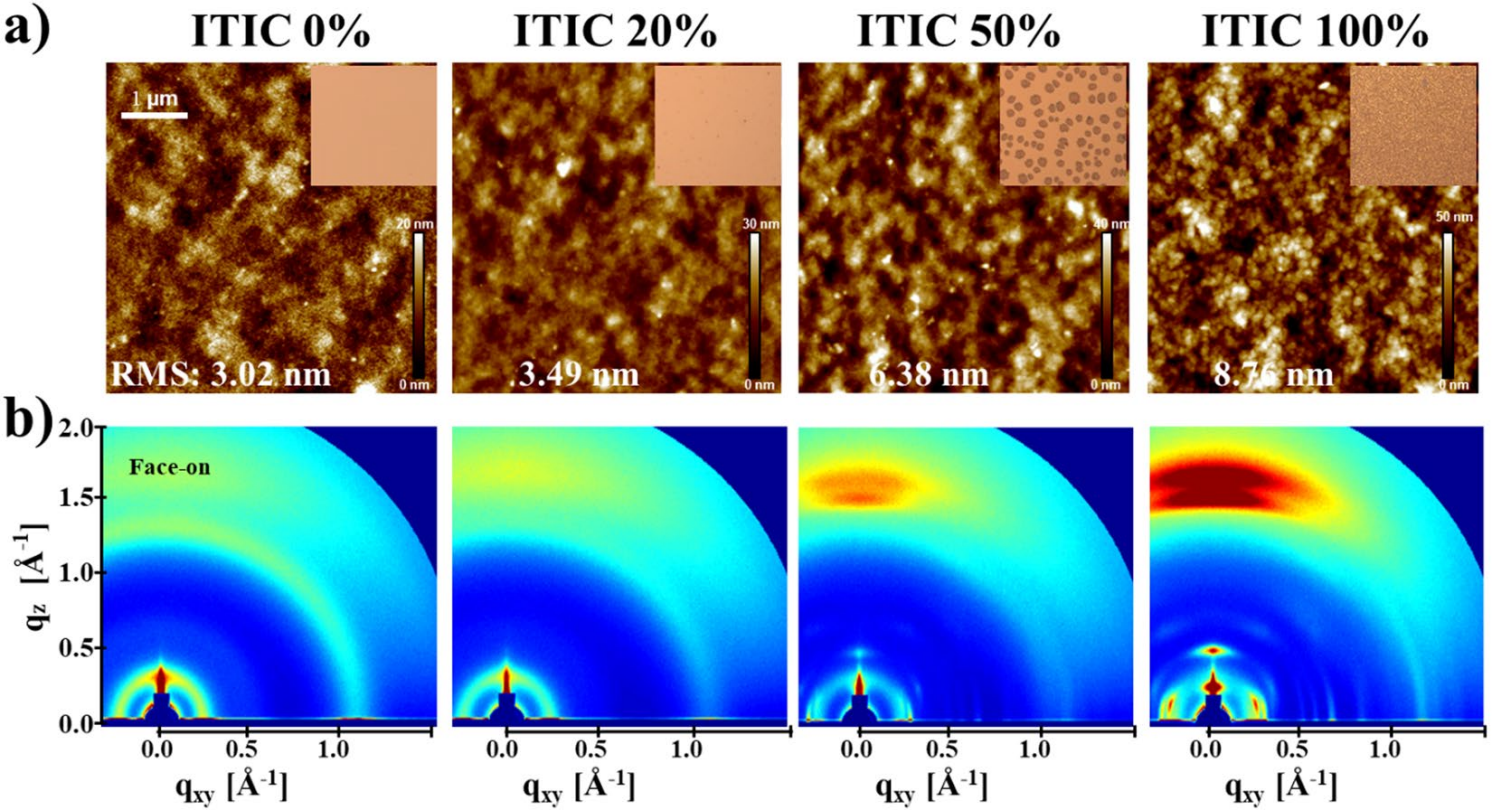
(**a**) AFM images and OM images (inset), and (**b**) GIWAXS data of PBT-OTT:ITIC:PC_71_BM blend films with different ITIC contents (wt%).

To investigate how the presence of ITIC affects the film morphologies and photovoltaic properties of the blends, atomic force microscopy (AFM) was used. The PBT-OTT:PC_71_BM films are homogeneous with a root-mean-square roughness (RMS) of 3.02 nm (Fig. 6a), which resulted from the high miscibility of PBT-OTT and PC_71_BM^32^. For [ITIC] = 20%, the morphology is aggregated (RMS = 3.49 nm), which enables the development of an interpenetrating network and reduces the interfacial trap density in the active layer, and thereby improves the PCE of the associated OSCs. Increases in [ITIC] result in the formation of large aggregated regions and surfaces with high RMS values. These effects produce a significant reduction in FF (Section 2.2).

To further understand the results for the morphologies, optical microscopy (OM) was conducted for various weight ratios of ITIC and PC_71_BM (Figs 6a and S2). For [ITIC] ≤ 20%, the resulting morphologies are almost clear, which indicates that the three components are well mixed. In contrast, obvious ITIC crystals form for [ITIC] > 30%; they are largest for [ITIC] = 40%, but decrease in size and increase in number for [ITIC] = 50% and 100%. For [ITIC] > 30%, the ITIC molecules do not mix well with PBT-OTT, so exciton dissociation is presumably inefficient and efficient electron transport pathways do not form.

To investigate the compatibility of PBT-OTT with ITIC and PC_71_BM, the surface energies of PBT-OTT, ITIC, and PC_71_BM were measured (Fig. S3; Table S5). Generally, the similar surface energies of components ensure good compatibility between the components. The obtained surface energies of PBT-OTT, ITIC, and PC_71_BM were 36.4, 47.7, and 37.3 mN m^−1^, respectively. The surface energy of PC_71_BM is closer to that of PBT-OTT than that of ITIC, i.e., PBT-OTT is more miscible with PC_71_BM than with ITIC. Hence, for [ITIC] > 30%, the phases separate and the film morphologies coarsen.

The crystal orientations and crystallite sizes of ternary blend films with various ITIC contents were studied by using grazing-incidence wide-angle X-ray scattering (GIWAXS) (Fig. 6b). The PBT-OTT:PC_71_BM film exhibits a face-on orientation, which can increase the favorability of intra- and inter-molecular charge carrier transport by the polymers in OSC devices^32^. The addition of ITIC at a concentration of 20% increases the intensity of the scattering peaks attributed to the face-on orientation. This preferential face-on orientation enhances charge transport and thereby the photovoltaic properties, as demonstrated by the improved SCLC results (Fig. S1). Further increases in [ITIC] up to 100% result in gradual increases in the intensity of the peak due to the face-on orientation; this trend indicates that the face-on orientation of ITIC favors the face-on orientation of PBT-OTT, i.e. the presence of ITIC enhances the intensity of the peak due to the face-on orientation. Furthermore, for [ITIC] ≥ 50%, two separate peaks due to face-on PBT-OTT and ITIC in PBT-OTT:ITIC binary blend films are evident in the GIWAXS data (Figs S4 and 6b), which indicates that the miscibility of PBT-OTT and ITIC is reduced.

The sizes of the crystal domains of PBT-OTT and ITIC in the blend films were compared by using the Scherrer equation^36^ to calculate the coherence lengths (CLs) (Table S6). Increases in [ITIC] from 40 to 100% result in gradually decreases in the CLs of ITIC. This trend is in agreement with the OM results in Fig. S4; for 0% ≤ [ITIC] ≤ 30%, the CLs of ITIC could not be calculated from the GI-WAX results, but the CLs of ITIC are expected to be small, as suggested by the OM results for 0% ≤ [ITIC] ≤ 30%. On the other hand, for 0% ≤ [ITIC] ≤ 20%, the CLs of PBT-OTT increase, but then decrease for 30% ≤ [ITIC]. As a result, the CLs of PBT-OTT mostly increase as the PC_71_BM content in the blends is increased, which indicates that an improvement in the degree of the molecular ordering of PBT-OTT is obtained. This effect may explain the increase in the intensity of the shoulder on the PBT-OTT peak adjacent to the maximum absorption peak in the UV absorption spectra of the film states (Fig. 2d). Although their enhanced face-on orientation is facilitated by the addition of ITIC, addition of ITIC at concentrations greater than 30% reduces the PCE due to the reduced miscibility of PBT-OTT and ITIC.

## Conclusion

We incorporated narrow-bandgap ITIC into the binary blend composed of wide-bandgap PBT-OTT and PC_71_BM. It was found that the addition of ITIC extends the light absorption of the active layer and increases photocurrent generation; it also establishes an energy level cascade with PBT-OTT and PC_71_BM, which promotes exciton dissociation and charge transfer. The optimum ITIC content (20%) results in a well-mixed and crystalline film morphology, which enhances the charge transport properties. Furthermore, the high-lying LUMO of ITIC, comparing with that of PC_71_BM, boosts the *V*_OC_ of the ternary OSCs. The low electron mobility of ITIC is compensated by the high electron mobility of PC_71_BM and balanced by the hole mobility of PBT-OTT. Therefore, charge recombination is effectively reduced and photo-generated charge carriers are efficiently collected at each electrode. The optimized ternary OSC with [ITIC] = 20% yields the highest PCE, 8.18%, which is 18% higher than that of the PBT-OTT:PC_71_BM binary OSC. These results confirm the usefulness of the ternary blend approach to the development of OSCs.

## Experimental Section

### Materials

PBT-OTT was synthesized by the methods used in our previous work^28^. The number-average molecular weight ($\bar{{Mn}}$) of the synthesized polymer was 22,000 g mol^−1^. All starting materials and reagents except for ITIC were purchased form Sigma-aldrich, Tokyo Chemical Industry Korea, Acros organics, and Frontier Scientific Inc., ITIC was purchased from Derthon Optoelectronic Materials Science Technology Co., LTD. All chemicals were used as received.

### Device fabrication

ITO substrate was washed with detergent, distilled water, acetone, and isopropyl alcohol sequentially with ultra-sonication for 20 min at each step. After UV-O_3_ treatment for 20 min, ZnO nanoparticles were deposited onto the ITO glass, evacuated for 4 hrs, and transferred into the N_2_-filled glove-box. PBT-OTT:PC_71_BM:ITIC blend solutions with controlled ratios were prepared in the chlorobenzene at 70 °C for overnight in the glove-box, and then deposited onto the ZnO-coated ITO glass. As a molecular additive, 1,8-diiodooctane (DIO) was added into the solution before depositing blend layer. The films were dried for 2 hrs. MoO_3_ (3 nm) and Au (60 nm) were thermally evaporated through a patterned mask.

### Characterization

All monomers synthesized in this work were characterized by ^1^H NMR (600 MHz) and ^13^C NMR (150 MHz) on a Bruker AVANCE III 600 spectrometer in chloroform-d solutions. The 1 H NMR chemical shift is shown in the d (ppm) unit relative to tetramethylsilane (TMS, d = 0) and refers to the peak signals corresponding to the non-deuterated remaining solvent. The polymers’ absorption spectrum was obtained by an UV spectrophotometer (UV-3220, Mecasys). AFM images were obtained using a MultiMode 8 Scanning Probe Microscope (VEECO Instruments Inc.) by tapping mode.

### Cyclic voltammetry analysis

The cyclic voltammetry (CV) data was obtained by using a PowerLab/AD instrument model system with the working electrode (glassy carbon disk), counter electrode (Pt wire), and reference electrode (Ag/Ag+) at a 50 mV s^−1^ potential scan speed in a solution of 0.1 M tetrabutylammonium hexafluorophosphate (n-Bu4NPF6)-anhydrous acetonitrile. Film was dropped from a 5.0 mg mL^−1^ warm CB solution onto the glassy carbon working electrode and dried before measurement under the nitrogen stream. With the use of the ferrocene/ferrocenium redox couple (Fc/Fc^+^), the potential of the Ag/AgCl reference electrode was internally calibrated. The HOMO energy level was calculated by using the equation; HOMO = −(4.80 + E_onset_).

## Supplementary information

Ternary Organic Solar Cells Based on a Wide-Bandgap Polymer with Enhanced Power Conversion Efficiencies
