# Supplementary material for: Ternary Organic Solar Cells Based on a Wide-Bandgap Polymer with Enhanced Power Conversion Efficiencies
Source: Sci Rep. 2019 Aug 19;9:12081. doi: 10.1038/s41598-019-48306-x (PMC6700133; doi:10.1038/s41598-019-48306-x)
Supplement: Supplementary file 1 — Ternary Organic Solar Cells Based on a Wide-Bandgap Polymer with Enhanced Power Conversion Efficiencies [file 41598_2019_48306_MOESM1_ESM.docx]

**Supporting Information**

**Ternary Organic Solar Cells Based on a Wide-Bandgap Polymer with Enhanced Power Conversion Efficiencies**

Hyeongjin Hwang,† Dong Hun Sin,† Chaneui Park,† and Kilwon Cho*

Department of Chemical Engineering, Pohang University of Science and Technology, Pohang, 790−784, Korea

[†] H. Hwang, D. H. Sin, and C. Park contributed equally to this work.

* Prof. Kilwon Cho, kwcho@postech.ac.kr


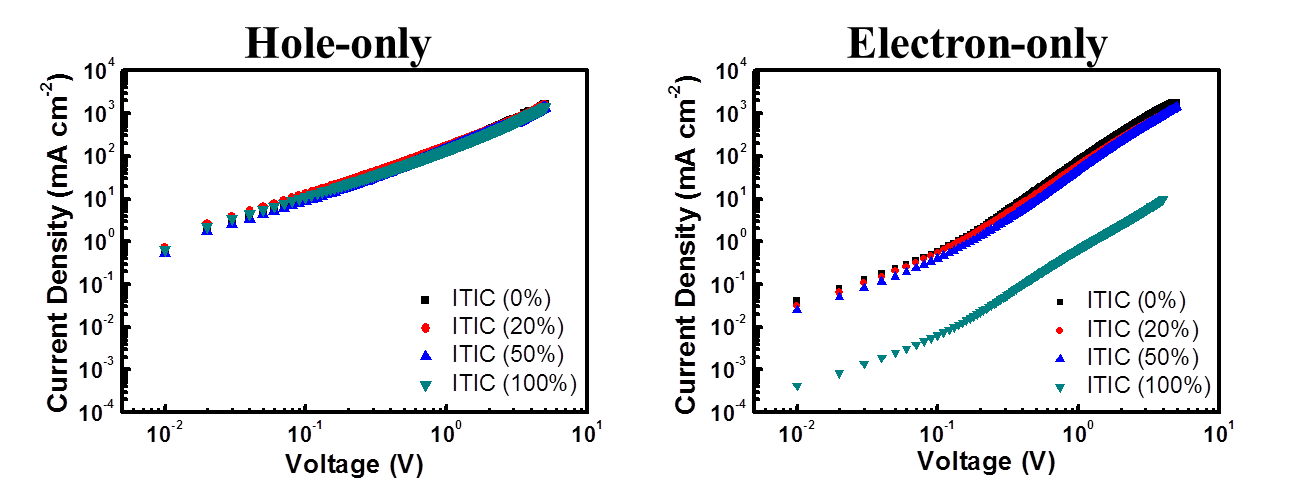


**Figure S1.** Dark current density versus effective voltage characteristics of a) hole-only devices (ITO/MoO_3_/blend/MoO_3_/Au) and b) electron only devices (Al/blend/LiF/Al), with photoactive layers with different PC_71_BM:ITIC ratio. In the case of electron-only devices the *V_bi_* =1.5 V is used.


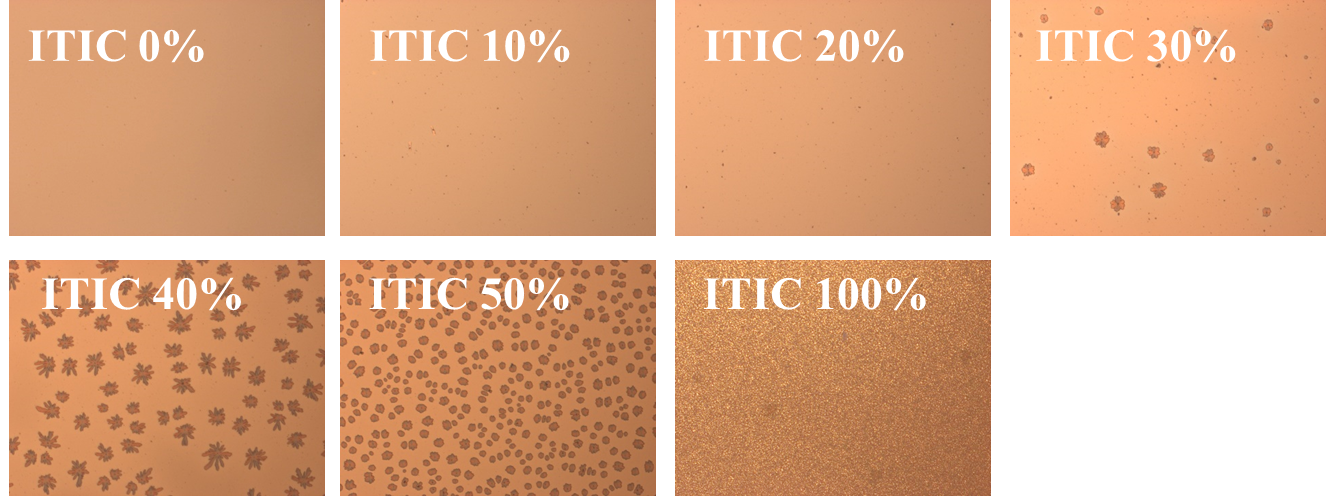


**Figure S2**. Optical microscopy of PBT-OTT:ITIC:PC_71_BM blend films with different ITIC contents (wt%).


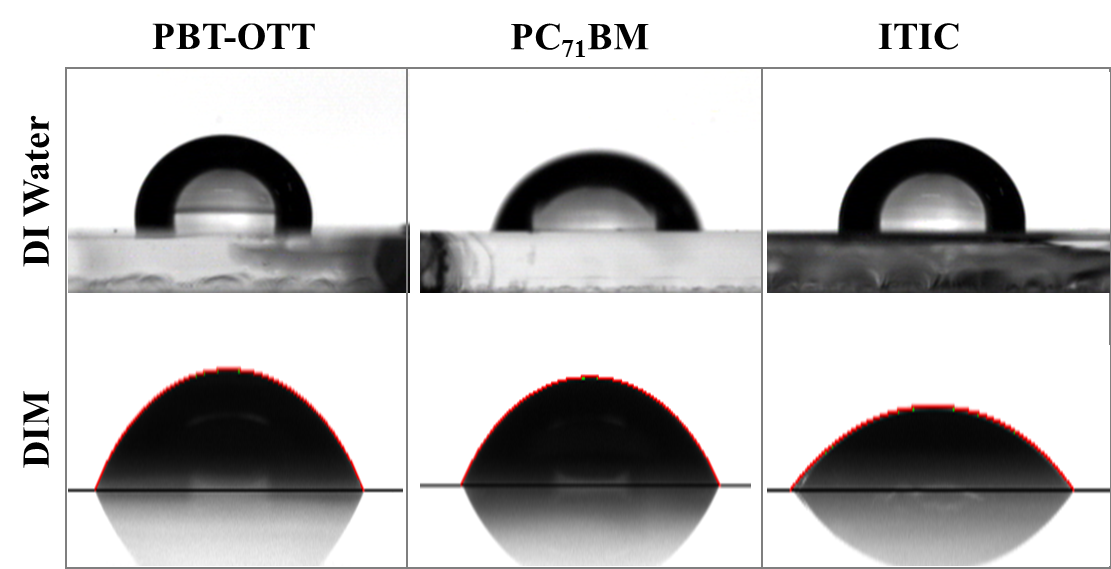


**Figures S3**. Contact angle images of PBT-OTT, PC_71_BM, and ITIC measured by using DI water and diiodomethane (DIM).


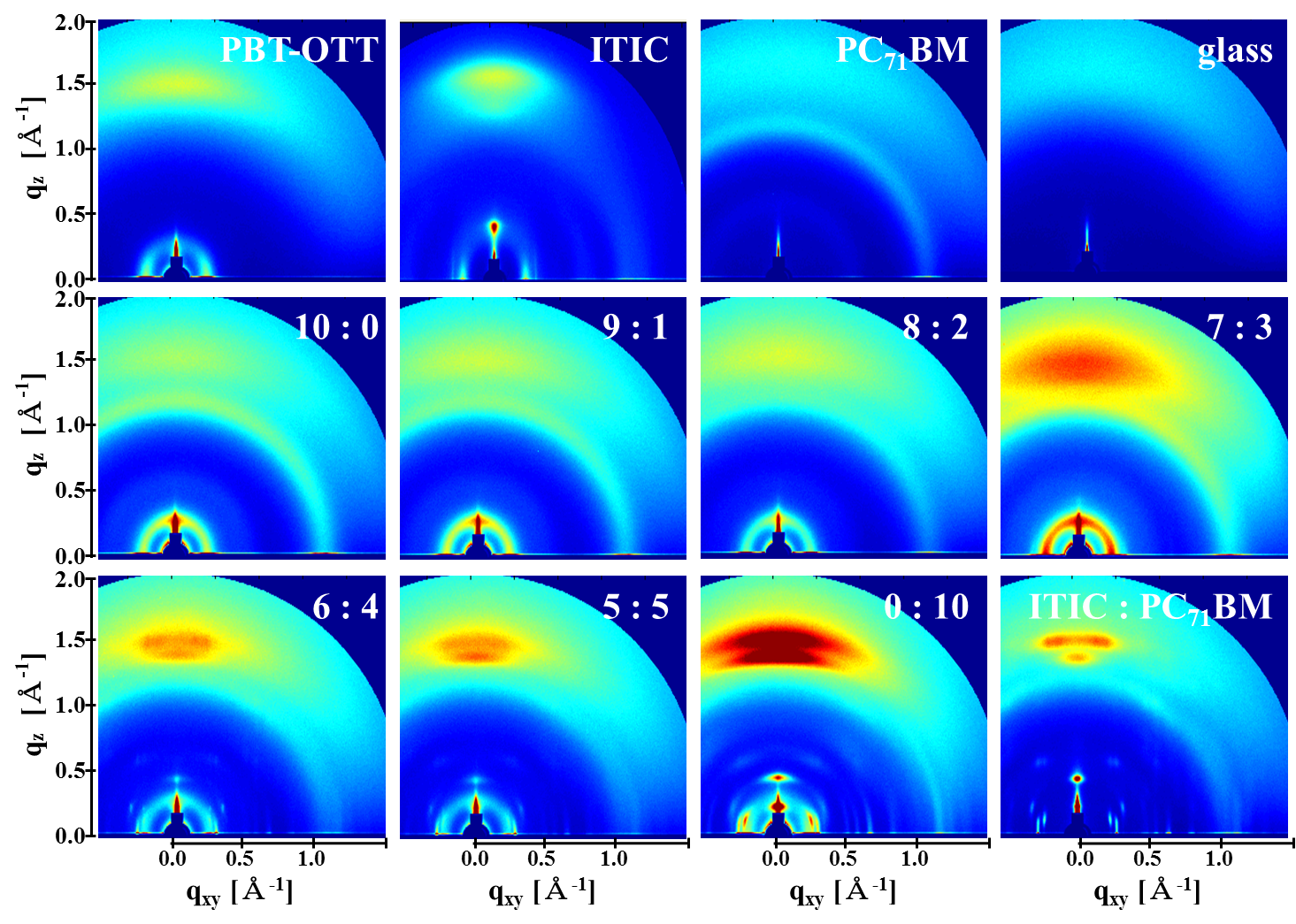


**Figure S4**. GIWAXS data of PBT-OTT:ITIC:PC_71_BM blend films with different ITIC contents.

**Table S1.** Photovoltaic parameters of ITIC:PC_71_BM binary device.

| PC_71_BM:ITIC  ratio | *J_SC_*  [mA cm^-2^] | *V_OC_*  [V] | FF  [%] | PCE  [%] |
| --- | --- | --- | --- | --- |
| 1:1 | 0.20 | 0.59 | 35 | 0.04 |

**Table S2**. Mobility results of space charge limited current (SCLC) devices with different ITIC contents.

| ITIC content (%) | *μ*_h_  [cm^2^/V·s] | *μ*_e_  [cm^2^/V·s] | *μ*_h_/*μ*_e_ |
| --- | --- | --- | --- |
| 0 | 1.91 x 10^-4^ | 3.13 x 10^-4^ | 1.64 |
| 20 | 2.24 x 10^-4^ | 2.17 x 10^-4^ | 0.97 |
| 50 | 1.81 x 10^-4^ | 1.92 x 10^-4^ | 1.06 |
| 100 | 1.86 x 10^-4^ | 2.22 x 10^-6^ | 0.01 |

**Table S3.** The results of maximum exciton generation and exciton dissociation probabilities with different ITIC contents.

| ITIC content (%) | *J_sat_*^a^  [A m^-2^] | *G_max_*^b^  [10^27^ m^-3^ s^-1^] | *P(E, T)* at short-circuit [%] |
| --- | --- | --- | --- |
| 0 | 152.3 | 9.51 | 88.7 |
| 20 | 165.1 | 10.31 | 90.5 |
| 50 | 116.8 | 7.29 | 74.7 |
| 100 | 145.4 | 9.08 | 79.5 |

^a)^ *J_sat_* = *qG_max_L*, ^b)^ *G_max_* : max exciton generation rate, and *J_ph_* = *qG_max_P(E, T)L*.

**Table S4.** The results of bimolecular recombination and trap-assisted recombination with different ITIC contents.

| ITIC content (%) | *J_SC_* slope | *V_OC_* slope [*k_b_T/q*] |
| --- | --- | --- |
| 0% | 0.975 | 1.66 |
| 20% | 0.990 | 1.44 |
| 50% | 0.896 | 1.76 |
| 100% | 0.914 | 1.81 |

**Table S5.** Contact angle values of PBT-OTT, PC_71_BM, and ITIC films and the corresponding surface energy calculated using the geometric mean equation, (1+cosθ)*γ*_pl_=2(*γ*_s_^d^*γ*_pl_^d^)^1/2^+2(*γ*_s_^p^*γ*_pl_^p^)^1/2^, where *γ*_pl_ and *γ*_s_ are the sample and probe liquid respectively.^1^ The PBT-OTT, PC_71_BM, and ITIC were dissolved in CB and spin-coated on the ITO glass at the condition of 1 wt% concentration, 1300 rpm for 90 s. Subsequently, the contact angle of each film was measured by using water and diiodomethane (DIM). From contact angle data, the surface tension of the polymers can be determined using the geometric mean equation which is commonly used to obtain the surface energy with two kinds of liquid where *θ_i_* is the droplet contact angle of water or DIM on the PBT-OTT, PC_71_BM, and ITIC films; *γ* ^total^ is the total surface tension; *γ* ^d^ and *γ* ^p^ are the dispersive and polar components of γ ^total^; γ^i^ is the total surface tension of the i material (i = water or DIM); *γ*_i_ ^d^ and *γ*_i_ ^p^ are the dispersive and polar components of *γ*_i._

|  | *θ ^water^*  [deg] | *θ ^DIM^*  [deg] | *γ* ^d^  [mN m^-1^ ] | *γ* ^p^  [mN m^-1^ ] | *γ* ^total^  [mN m^-1^ ] |
| --- | --- | --- | --- | --- | --- |
| PBT-OTT | 99 | 47.9 | 0.1 | 36.4 | 36.4 |
| ITIC | 93 | 25.0 | 0.1 | 47.6 | 47.7 |
| PC_71_BM | 76 | 48.6 | 7.4 | 29.9 | 37.3 |

**Table S6.** The coherence length (CL) of PBT-OTT and ITIC in ternary blend films of different ITIC contents.

| ITIC (wt %) |  | 0 | 10 | 20 | 30 | 40 | 50 | 100 |
| --- | --- | --- | --- | --- | --- | --- | --- | --- |
| CL/Å^-1^ | PBT-OTT | 61 | 68 | 69 | 66 | 66 | 63 | 50 |
|  | ITIC | N/A | N/A | N/A | N/A | 307 | 263 | 187 |

**References**

1 Park, J. H. *et al.* Enhanced device performance of organic solar cells via reduction of the crystallinity in the donor polymer. *J Mater Chem* **20**, 5860-5865 (2 010).
